# Gasdermin D-deficient mice are hypersensitive to acute kidney injury

**DOI:** 10.1038/s41419-022-05230-9

**Published:** 2022-09-15

**Authors:** Wulf Tonnus, Francesca Maremonti, Alexia Belavgeni, Markus Latk, Yoshihiro Kusunoki, Anne Brucker, Anne von Mässenhausen, Claudia Meyer, Sophie Locke, Florian Gembardt, Kristina Beer, Paul Hoppenz, Jan U. Becker, Christian Hugo, Hans-Joachim Anders, Stefan R. Bornstein, Feng Shao, Andreas Linkermann

**Affiliations:** 1 Department of Internal Medicine 3, University Hospital Carl Gustav Carus at the Technische Universität Dresden, Dresden, Germany; 2 Biotechnology Center, Technische Universität Dresden, Dresden, Germany; 3 Renal Division, Department of Medicine IV, University Hospital of the Ludwig Maximilian University, Munich, Germany; 4 Institute of Pathology, University Hospital of Cologne, Cologne, Germany; 5 Diabetes and Nutritional Sciences, King’s College London, London, UK; 6 Center for Regenerative Therapies, Technische Universität Dresden, Dresden, Germany; 7 Paul Langerhans Institute Dresden of Helmholtz Centre Munich at University Clinic Carl Gustav Carus of TU Dresden Faculty of Medicine, Dresden, Germany; 8 Lee Kong Chian School of Medicine, Nanyang Technological University, Singapore, Singapore; 9 National Institute of Biological Sciences (NIBS), Beijing, China

**Keywords:** Cell death, Pathogenesis, Acute kidney injury

## Abstract

Signaling pathways of regulated necrosis, such as necroptosis and ferroptosis, contribute to acute kidney injury (AKI), but the role of pyroptosis is unclear. Pyroptosis is mediated by the pore-forming protein gasdermin D (GSDMD). Here, we report a specific pattern of GSDMD-protein expression in the peritubular compartment of mice that underwent bilateral ischemia and reperfusion injury (IRI). Along similar lines, the GSDMD-protein expression in whole kidney lysates increased during the first 84 h following cisplatin-induced AKI. Importantly, unlike whole kidney lysates, no GSDMD-protein expression was detectable in isolated kidney tubules. In IRI and cisplatin-induced AKI, GSDMD-deficient mice exhibited hypersensitivity to injury as assessed by tubular damage, elevated markers of serum urea, and serum creatinine. This hypersensitivity was reversed by a combined deficiency of GSDMD and the necroptosis mediator mixed lineage kinase domain-like (MLKL). In conclusion, we demonstrate a non-cell autonomous role for GSDMD in protecting the tubular compartment from necroptosis-mediated damage in IRI.

## Introduction

Acute kidney injury (AKI) emerges as a global health burden [1]. To allow the development of novel therapeutic strategies to treat AKI [2, 3] and prevent AKI-to-CKD (chronic kidney disease) transition [4], molecular insights into the mechanisms of failed tubular recovery, nephron loss and acute tubular necrosis (ATN) are required to allow future prevention medicine in those patients [3].

Regulated necrosis is mediated by a diverse set of cell death pathways [5]. In general, two separate systems have been delineated. Apoptosis, a caspase-regulated nonimmunogenic cell death that is not associated with the rupture of the plasma membrane [6], is tightly interconnected to two types of regulated necrotic cell death referred to as necroptosis [7, 8] and pyroptosis [9–14]. Necroptosis is mediated by kinases, such as RIPK1 [15, 16] and RIPK3 [17–19], and is executed by MLKL [20, 21] and counteracted by the ESCRT-III complex [22, 23]. Pyroptosis occurs downstream of inflammasome activation and is mediated by caspase-1/11-dependent cleavage of gasdermin D (GSDMD) [14, 24, 25]. N-terminal fragments of GSDMD were demonstrated to oligomerize and insert into the plasma membrane to mediate the loss of membrane integrity [25–28]. Importantly, pyroptosis, apoptosis, and necroptosis (jointly also referred to as PANoptosis [29]) exhibit a complex system of host defense against viruses [30] and bacteria [31].

In contrast to the signaling system of pyroptosis, apoptosis, and necroptosis, a separate system exists referred to as ferroptosis, death by lipid peroxidation following iron-catalyzed Fenton reactions [32, 33]. In contrast to other pathways, cell death during ferroptosis was demonstrated to follow intercellular osmotic patterns of cell death propagation [34–37], thereby affecting functional units, such as the synchronized death of renal tubules [38], rather than individual cells.

In acute kidney injury models, such as cisplatin-induced AKI and ischemia-reperfusion injury, the roles of ferroptosis, apoptosis, and necroptosis have been subject to extensive investigations over the last decades [39–41]. However, the role of pyroptosis in AKI was hardly explored, and the limited existing results are controversial [42–46].

Here, we demonstrate a unique peritubular pattern of GSDMD expression during AKI and show GSDMD-deficient mice to be hypersensitive to IRI and cisplatin-induced AKI. Mechanistically we identify this hypersensitization to be mediated by MLKL-dependent necroptosis.

## Results

### Ischemia-reperfusion injury induces gasdermin D (GSDMD) expression in the peritubular compartment of necrotic segments

**Fig. 1:**
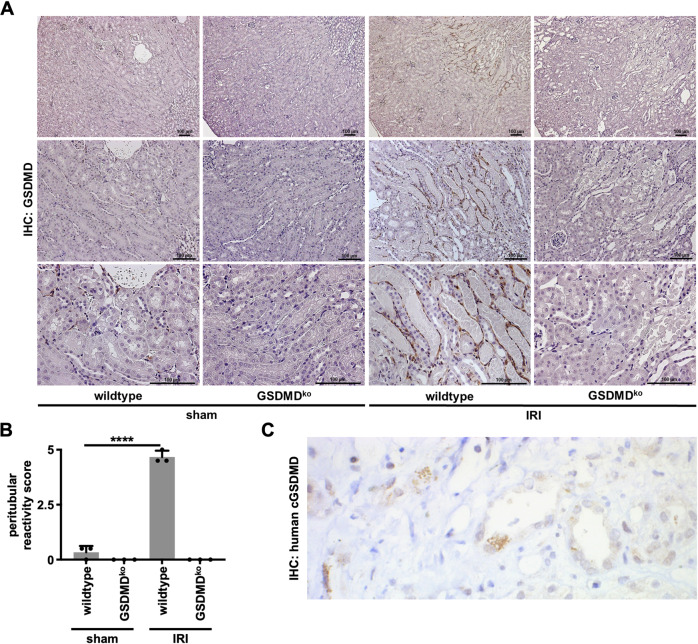
Gasdermin D-positivity in murine and human renal AKI. Eight- to twelve-week-old male wild-type and *Gsdmd*-deficient mice underwent severe IRI and were evaluated after 48 h. **A** Immunohistochemistry (IHC) for GSDMD reveals specific peritubular positivity lining necrotic tubules. **B** Quantification of peritubular GSDMD reactivity. (*n* = 3 mice/group) (Student’s *t*-test for ****p < 0.0001). **C** Human biopsy IHC for cleaved gasdermin D in an AKI patient sample.

GSDMD was identified as the major downstream mediator of pyroptosis. We, therefore, stained sections obtained from male C57Bl/6N mice that underwent bilateral renal ischemia-reperfusion injury (IRI) for GSDMD by immunohistochemistry. In order to detect potential off-target staining of the antibody, *Gsdmd*-deficient mice served as controls (Fig. 1A and Fig. S1). While the baseline expression of GSDMD appeared minimal, we observed a strictly peritubular staining pattern of GSDMD within 48 h following the onset of reperfusion. This pattern appeared particularly dominant next to areas of extensive acute tubular necrosis in the S3 segment of the proximal tubule, while no signal at all was detected in the sections obtained from *Gsdmd*-ko mice (Fig. 1B). Additionally, the cleaved N-terminal fragment of GSDMD was detectable in indication biopsies of patients suffering from AKI (Fig. 1C).

To investigate the pyroptotic proteins in AKI, we generated *Gsdmd*- and gasdermin E (*Gsdme*)-deficient mice and crossed them to obtain *Gsdmd/Gsdme* double-deficient littermates. Among untreated animals of these genotypes, the investigation of renal tissue by periodic acid-Schiff (PAS) staining revealed no obvious histological signs of tubular damage or any detectable phenotype (Fig. S2A). In line with this observation, the concentrations of both serum creatinine (Fig. S2B) and serum urea (Fig. S2C) were indistinguishable from wild-type littermates.

### Genetic deficiency of GSDMD, GSDME, or combined deficiency hypersensitizes mice to IRI-induced AKI

**Fig. 2:**
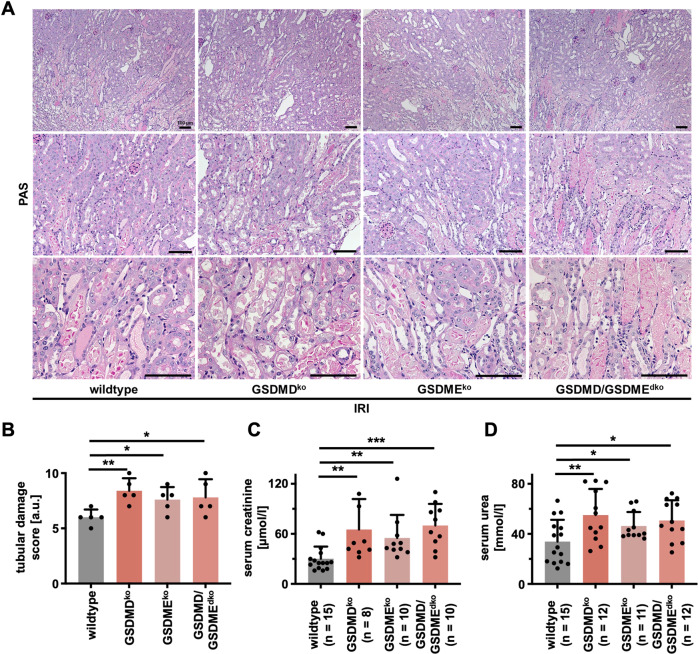
Deficiency of gasdermin D, gasdermin E, or the combined deficiency sensitize to ischemia-reperfusion-induced acute kidney injury. Eight- to twelve-week-old male wild-type, *Gsdmd*^ko^, *Gsdme*^ko^, and *Gsdmd*/*Gsdme*^dko^ mice underwent moderate IRI and were sacrificed after 48 h of reperfusion. **A** Histological evaluation revealed increased tubular damage in comparison to wild-type mice, quantified by tubular damage scores (**B**). Serum creatinine (**C**) and serum urea (**D**) concentrations upon sacrifice 48 h after the onset of reperfusion. Note the reduced kidney function in *Gsdmd*^ko^, *Gsdme*^ko^, and *Gsdmd*/*Gsdme*^dko^ mice. (*n* = 8–15 mice/group) (Student’s *t*-test for *p < 0.05, **p < 0.01, ***p < 0.001).

We investigated *Gsdmd-*, *Gsdme-,* and double-deficient mice in a standard model of moderate bilateral IRI (compare methods section for details). In comparison to wild-type mice, significantly aggravated necrotic regions in the S3 segment of the proximal tubules and the outer stripe of the inner medulla, as detected by PAS staining, were observed (Fig. 2A, B). In keeping with this observation, we discovered a significantly larger increase in serum concentrations of creatinine (Fig. 2C) and urea (Fig. 2D) in *Gsdmd*- and *Gsdme*-deficient mice compared to wild-types. Similar values were detected upon combined deficiency of GSDMD and GSDME. Clinically, the ischemic dose that leads to nephron loss may vary considerably between individuals. We, therefore, added a set of separate experiments with an increased dose of ischemia before reperfusion, referred to as severe IRI. However, no significant differences between wild-type and *Gsdmd*-deficient mice were detectable after 24 h or 48 h by PAS staining (Fig. S3A, B) or functional markers of renal injury (Fig. S3C, D). In addition, we detected no significant difference in post-IRI numbers of infiltrating CD3 cells (Fig. S4A, B) upon assessment of severe IRI. In summary, these results suggest that the genetic deficiency of GSDMD correlates with the loss of a protective non-cell autonomous signal that aggravates the outcome following IRI in mice.

**Fig. 3:**
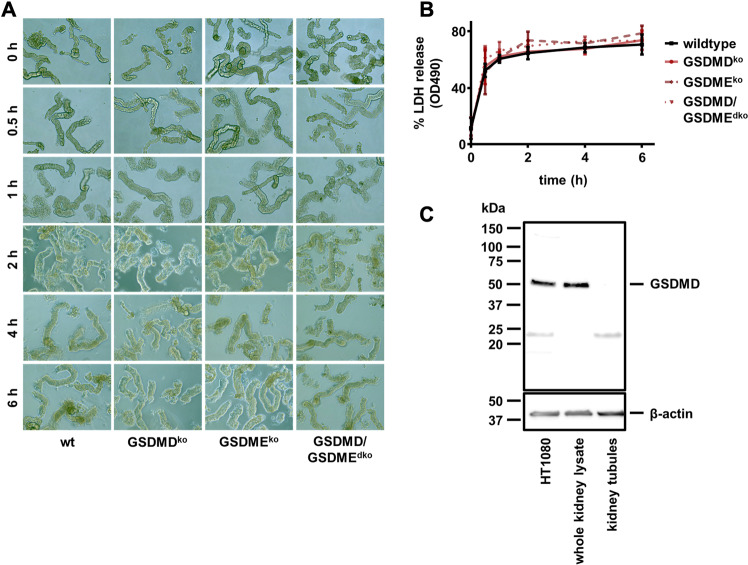
Gasdermin D is not detectable in murine kidney tubules and is not involved in tubular necrosis. **A** Still images of freshly isolated tubules from male wild-type, *Gsdmd*^ko^, *Gsdme*^ko^, and *Gsdmd*/*Gsdme*^dko^ mice at indicated time points (0 h until 6 h) after preparation. **B** LDH release into the medium was quantified over time without significant differences. **C** Western blot of murine GSDMD. Note the absence of the GSDMD band in lysates from freshly isolated renal tubules. (*n* = 2 independent experiments/group).

As demonstrated above, immunohistochemistry demonstrated no tubular expression of GSDMD (Fig. 1A). Therefore, we hypothesized that the tubular injury may develop secondarily and independently of GSDMD. To test this hypothesis, we isolated renal tubules from *Gsdmd-*, *Gsdme-,* and double-deficient littermates and investigated morphological changes over 6 h. As demonstrated in Fig. 3A, no major morphological differences were observed between these groups. Along similar lines, the LDH released from these tubules exhibited no significant differences between the groups (Fig. 3B). Importantly, and in line with the previous observation, we failed to detect GSDMD by western blotting from lysates of kidney tubules, while a clear band at the expected size appeared in control HT1080 cells and whole kidney lysates (Fig. 3C). These results suggest a lack of GSDMD expression in the tubular compartment.

### Hypersensitization to IRI in *Gsdmd*-deficient mice is reversed by combined genetic deletion of the necroptosis-executor mixed lineage kinase domain-like (MLKL)

**Fig. 4:**
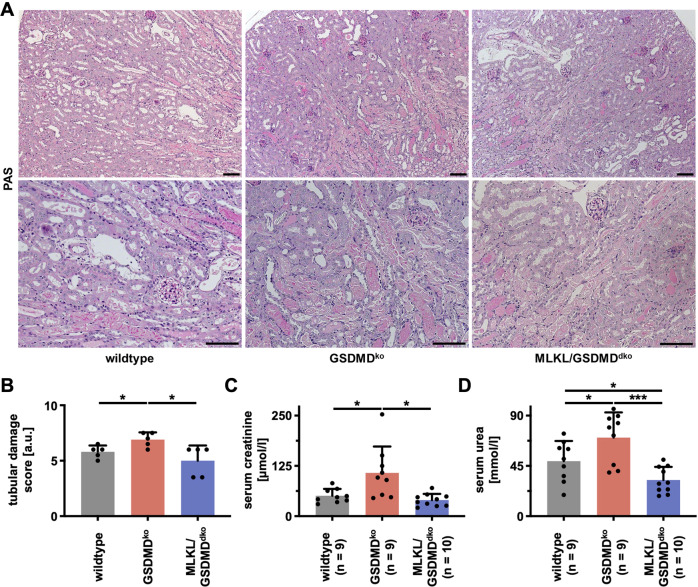
Co-deletion of the necroptosis-executor MLKL reverses hypersensitivity toward IRI of *Gsdmd*-deficient mice. Male 8–12-week-old wild-type, *Gsdmd*^ko^, and *Mlkl/Gsdmd*^dko^ mice underwent moderate IRI. **A**, **B** At 48 h after the onset of reperfusion, histology demonstrated reduced tubular damage in *Mlkl/Gsdmd*^dko^ mice as quantified by tubular damage score. **C**, **D** Co-deletion of MLKL also significantly decreased serum concentrations of creatinine and urea. (*n* = 9–10 mice/group) (Student’s *t*-test for *p < 0.05; **p < 0.01; ***p < 0.001).

It has been demonstrated by us and others that pyroptosis and necroptosis are interconnected and cross-regulated signaling pathways of regulated necrosis [31, 47–49]. We, therefore, speculated that *Gsdmd*-deficiency might activate the necroptosis pathway, which we previously demonstrated to be involved in AKI at the level of RIPK1 [50], RIPK3 [51], MLKL [52], and ESCRT-III [22]. Thus, we crossed *Gsdmd*-deficient mice with MLKL-deficient mice in our facility and observed no spontaneous histological and functional differences between wild-type, *Gsdmd*-ko and *Mlkl*/*Gsdmd*-dko kidneys (Fig. S5A–C). In line with our hypothesis, the co-deficiency of MLKL in addition to GSDMD reversed the increased tubular morphological changes observed in GSDMD-ko mice challenged with IRI (Fig. 4A, B). Furthermore, serum concentrations of creatinine (Fig. 4C) and urea (Fig. 4D) in *Mlkl*/*Gsdmd*-dko mice were kept at the level of wild-type mice in this model. These data suggest an in vivo interplay between pyroptosis and necroptosis.

### A GSDMD-to-MLKL switch mechanism hypersensitizes *Gsdmd*-deficient mice to cisplatin-induced renal injury

**Fig. 5:**
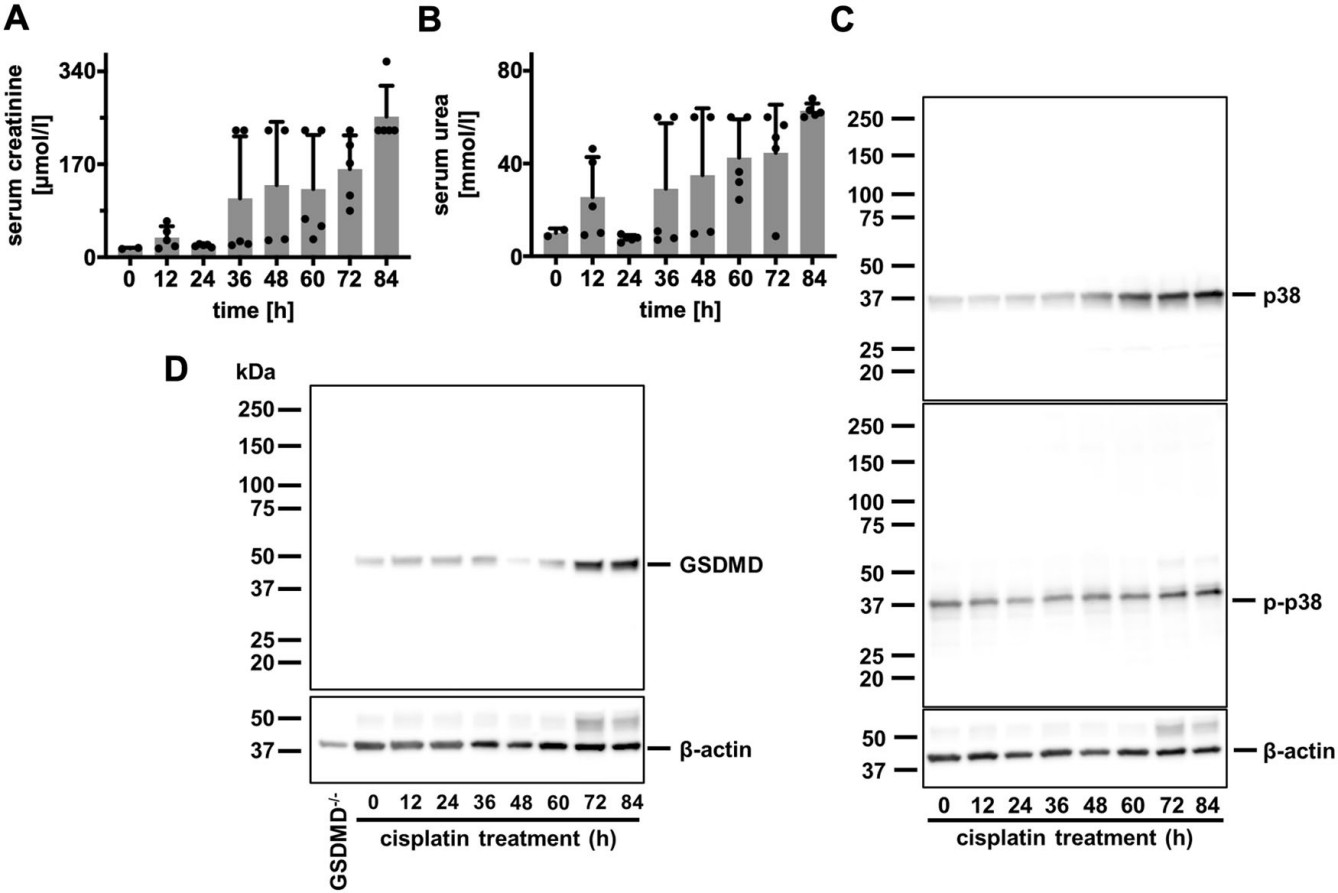
Gasdermin D-positivity increases over time in cisplatin-induced AKI. Eight- to twelve-week-old female wild-type mice (*n* = 5 mice/time point) were treated with 20 mg/kg body weight cisplatin intraperitoneally. **A**, **B** At indicated time points, mice were sacrificed and serum concentrations of creatinine and urea were measured. **C** Corresponding western blot analysis of whole kidney lysates stained for p38 MAP kinase and phospho-p38 MAP kinase (p-p38). **D** Corresponding Western Blot analysis of whole kidney lysates demonstrates increased GSDMD expression over time.

To investigate the role of GSDMD in a distinct AKI model, we subjected wild-type mice to intraperitoneal injection of 20 mg/kg body weight cisplatin (cisplatin-induced AKI) and separated the mice into eight distinct groups. Mice were sacrificed after 0, 12, 24, 36, 48, 60, 72 and 84 h. Serum levels of creatinine (Fig. 5A) and urea (Fig. 5B) were obtained at these time points, and whole kidney lysates were generated following euthanasia. We previously demonstrated the time course of p38 and phospho-p38 MAP kinase activation in renal tissue following IRI [38]. As demonstrated in Fig. 5C, D, we observed a comparable pattern of the induction of GSDMD-protein expression within 84 h.

**Fig. 6:**
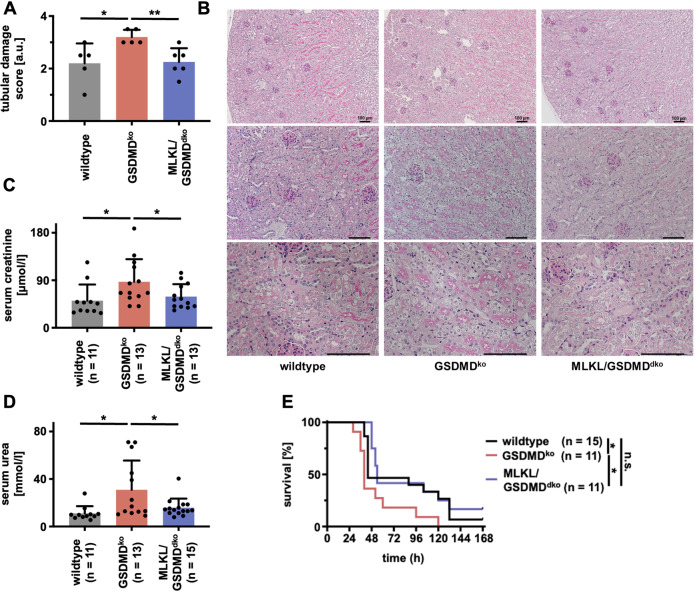
Co-deletion of MLKL reverses the hypersensitivity of *Gsdmd*-deficient mice in cisplatin-induced AKI. Female 8–12-week-old wild-type, *Gsdmd*^ko^, and *Mlkl/Gsdmd*^dko^ mice were treated with 20 mg/kg body weight of cisplatin i.p. **A**, **B** After 48 h, mice were sacrificed, histological sections were stained for PAS, and tubular damage was quantified. Note the deterioration of tubular damage in *Gsdmd*^ko^-mice compare to wild-type mice, which was reversed in *Mlkl/Gsdmd*^dko^ mice. **C**, **D** Serum concentrations of creatinine and urea at 48 h after the onset of reperfusion in indicated groups. **E** Kaplan–Meier survival plot of indicated groups of mice following a single injection of cisplatin. Note the significant survival benefit between the *Gsdmd*^ko^-mice and *Mlkl/Gsdmd*^dko^-mice. The latter are no different from wild-type mice. (*n* = 11–15 mice/group) (Student’s *t*-test or log-rank test with *p < 0.05, **p < 0.01).

We next compared wild-type mice alongside *Gsdmd*-ko and *Mlkl*/*Gsdmd*-dko mice in the model of cisplatin-induced AKI. As observed in the model of IRI, *Gsdmd*-ko were hypersensitive to tubular damage 48 h after injection (Fig. 6A, B), and functional markers of AKI were significantly increased (Fig. 6C, D). Again, genetic co-deletion of MLKL reversed these features to the level of wild-type mice (Fig. 6A, D). Finally, we compared the overall survival of wild-type, *Gsdmd*-ko, and *Mlkl*/*Gsdmd*-dko following cisplatin injection (Fig. 6E). While *Gsdmd*-ko mice succumbed significantly earlier than wild-type mice, the survival of *Mlkl*/*Gsdmd*-dko mice was comparable to wild-type mice.

The mouse model of cisplatin-mediated AKI was repeatedly demonstrated to depend on a TNFα-signal, and interference with TNF (e.g., by etanercept) is known to partially protect mice in this model [53–55]. In addition, *Gsdmd*-deficient mice are resistant to the TNFα-induced SIRS model [10]. To re-evaluate published data on cisplatin-induced AKI, we reduced the cisplatin dose to 15 mg/kg body weight. While in wild-type littermates, the effect of etanercept was solidly reproducible, and *Gsdme*-deficient mice exhibited no significant difference in this model (Fig. S6A), we detected no significant difference through the addition of etanercept in *Gsdmd*-deficient mice (Fig. S6B). Together with previously published data on the role of GSDMD in the TNFα-induced SIRS model [10], our data indicate that, again, distinct TNFα-dependent effects rely on GSDMD.

### *Gsdmd*-deficient mice are hypersensitive to calcium oxalate-induced chronic nephropathy

To test a third model of subacute AKI that transfers into chronic kidney disease (CKD), we investigated the effects of *Gsdmd*-deficient mice in a mouse model of AKI induced by calcium oxalate (CaOx). The glomerular filtration rate (GFR) measured at 20 days following the onset of a CaOx-enriched diet (see methods section for details) showed a significant decrease only in the *Gsdmd*-deficient mice (Fig. S7A). In keeping with this observation, the functional kidney markers of serum creatinine and urea (BUN) were increased (Fig. S7B, C), although this effect did not reach statistical significance. Importantly, the mRNA levels of the kidney tubular damage marker NGAL were indistinguishable (Fig. S7D). Collectively, these data indicate that genetic deficiency of the pyroptosis executor protein GSDMD sensitizes to acute kidney injury in three independent rodent models.

## Discussion

Acute kidney injury is known to be partially mediated by necroptosis and ferroptosis, two signaling pathways of regulated necrosis [39]. The NLRP3 inflammasome [56, 57] and GSDMD-mediated pyroptosis [47] were demonstrated to comprise common regulatory motifs with necroptosis [58]. In this sense, GSDMD is regulated and can be cleaved by caspase-8 [10, 59–62]. Hence, we did not directly address the question of which protease processes GSDMD in our specific models. Also, in response to virally infected cells, necroptosis and pyroptosis regulate an intertwined system [63, 64]. It has recently been demonstrated that macrophages carrying a Lrrk2G2019S mutation display plasticity between pyroptosis and necroptosis, which is mediated by translocation of cGSDMD to mitochondria to form ROS-releasing pores [65]. The release of mitochondrial ROS was postulated to promote necroptosis by increased phosphorylation of RIPK3. However, it remains unclear whether such plasticity exists in wild-type macrophages and how mitochondrial ROS might lead to the phosphorylation of RIPK3. To the best of our knowledge, crosstalk between necroptosis and pyroptosis has not been investigated in AKI. In fact, we previously investigated an interference with mitochondrial permeability transition (*ppif*-deficiency) alongside the induction of necroptosis and reported that these are distinct pathways that are activated in parallel during AKI [51]. Recently, we demonstrated ROS scavenging by lipophilic radical trapping agents to increase rather than reduce the phosphorylation of RIPK1 and MLKL upon necroptosis induction in HT29 cells [66]. Therefore, our data do not support a “pyroptosis-to-necroptosis switch” within a single cell, such as a macrophage, to be of significant relevance to AKI models. Here, however, we demonstrate the first evidence of pyroptosis and necroptosis to function in different cell types during AKI to prevent further deterioration and propagation of tubular damage. Rather than being primarily involved in renal tubular epithelial cell death, GSDMD-positive cells may constrain necroptotic cell death in the tubules. However, the exact mechanism of these functions remains to be elucidated in future studies.

Recently, the FDA-approved drug disulfiram was demonstrated to inhibit pyroptosis by interfering with the pore-forming activity of cleaved GSDMD [67]. Although, to the best of our current knowledge, disulfiram has not been investigated in AKI models, it has been demonstrated to augment oxidative stress in rat brains following bilateral carotid artery occlusion [68]. Our data that point to a non-cell autonomous tissue-protective function of GSDMD are in line with those discoveries. However, two recent publications suggested that the genetic deficiency of GSDMD or the inhibition of the NLRP3 inflammasome may be protective in models of AKI and protect from tubular necrosis [42, 43]. Our data are in contrast to those publications and in line with post-IRI single-cell analysis that failed to detect GSDMD expression in tubular epithelial cells following murine kidney IRI [69]. Interestingly, another paper demonstrated an increase of the N-terminal fragment of GSDMD over the time course of 48 h after IRI [70], which is in line with our results from both IRI and cisplatin-induced AKI. A possible explanation for the different conclusions may be the lower number of mice per group [42, 43, 70] and the differences in severities of the AKI models [42, 43, 70]. In this regard, we consider it particularly important to reconcile the absence of GSDMD expression in renal tubules (Fig. 1A and Fig. 3C).

In conclusion, we demonstrated GSDMD to be upregulated in the peritubular compartment, specifically next to necrotic tubular epithelial cells, to function as a suppressor of AKI by a previously unknown non-cell autonomous crosstalk to the necroptosis machinery. These data add yet another layer of complexity to the pathogenesis of AKI.

## Materials and methods

### Cells and reagents

The human HT1080 cell line was purchased from the American Type Culture Collection. Cells were cultured in a humidified 5% CO_2_ atmosphere and cultured in Dulbecco’s modified Eagle medium (DMEM, Thermo Fisher) supplemented with 10% (v/v) FBS (Thermo Fisher), 100 U/ml penicillin, and 100 μg/ml streptomycin (Pen/Strep, Thermo Fisher).

### Western blotting

Cells were lysed in ice-cold 10 mM Tris-HCl, pH 7.5, 50 mM NaCl, 1% Triton X-100, 30 mM sodium pyrophosphate, 50 mM NaF, 100 μM Na_3_VO_4_, 2 μM ZnCl_2_, and 1 mM phenylmethylsulfonyl fluoride (PMSF, modified Frackelton buffer) for 30 min on ice. Insoluble material was removed by centrifugation (14,000 × *g*, 30 min, 4 °C). Protein concentration was determined using a commercial Bradford assay kit according to the manufacturer’s instructions (Thermo Fisher). Equal amounts of protein were resolved on a 4–15% gradient SDS/PAGE gel and transferred to a PVDF membrane (BIO-RAD). Mouse kidneys were cut in half immediately after the sacrifice of the mice and placed in tubes containing beads and ice-cold modified Frackelton buffer. The tubes were transferred to a bead mill homogenizer, and samples were homogenized. Equal amounts of protein (typically 35 μg per lane) were incubated with Roti-Load 1 (ROTH), resolved on a 4–15% gradient SDS/PAGE gel, and transferred to a PVDF membrane (BIO-RAD). Isolated primary murine tubules (see Isolation of primary murine renal tubules section) were incubated with Roti-Load 1 (ROTH) and resolved on a 4–15% gradient SDS/PAGE gel and transferred to a PVDF membrane (BIO-RAD). Primary antibody incubation was performed for GSDMD (Abcam, ab219800), p38 MAPK (Cell Signaling, Cat# 9212), phospho-p38 MAPK (Cell Signaling, Cat# 4511), and anti-β-Actin (Cell Signaling, Cat# 3700 S) at concentrations 1:1000. Secondary antibodies (anti-mouse, HRP-linked antibody, Cell Signaling, Cat# 7076 S; anti-rabbit, HRP-linked antibody, Cell Signaling, Cat# 7074 S) were applied at concentrations of 1:5000. Proteins were then visualized by enhanced chemiluminescence (ECL; Amersham Biosciences). All data presented in the manuscript are representative examples of at least three biological replicates of the specific experiment.

### Mice

All mice referred to as wild-types (WT) in this study are C57Bl/6N obtained from Charles River Laboratories (Sulzfeld, Germany). *Gsdmd*-deficient mice [24] and *Gsdme*-deficient mice [71] were described earlier and crossed to generate GSDMD/GSDME^dko^ mice. GSDMD^ko^ mice were additionally crossed to *Mlkl*^ko^ mice, kindly provided by James Murphy [72], to generate *Mlkl/Gsdmd*^dko^ mice. To minimize background effects, *Mlkl/Gsdmd*^dko^ mice were interbred for at least 10 generations before including them in experiments. All in vivo experiments were performed according to the Protection of Animals Act, after approval of the German local authorities in Dresden and Munich (TVV57/2017). Group sizes were calculated beforehand with the help of a professional statistician from the Institute of Medical Informatics and Biometry of the Technische Universität Dresden. In all experiments, mice were rigorously matched for age, sex, weight, and genetic background and used as indicated in each individual experiment. Thus, all genetically modified mice used in experiments were genotyped by PCR, and investigators were strictly unaware of the genotype and experimental groups until data collection was completed.

### Murine ischemia/reperfusion injury (IRI)

The technique has been previously described in detail [73]. Concisely, 8–12-week-old male mice were co-housed at 2–5 mice/ cage in IVCs in our facility under 12 h light circles. Induction of kidney IRI was performed via a midline abdominal incision and a bilateral renal pedicle clamping for either 36 min (severe IRI) or 30 min (moderate IRI) using microaneurysm clamps (FST). Throughout the surgical procedure, the body temperature was maintained between 36 °C and 37 °C by continuous monitoring using a temperature-controlled self-regulated heating system (Fine Science Tools). After the removal of the clamps, reperfusion of the kidneys was confirmed visually. The abdomen was closed in two layers using standard 6-0 sutures. Sham-operated mice underwent identical surgical procedures, except that microaneurysm clamps were not applied. To maintain fluid balance, all mice were supplemented with 1 mL of prewarmed PBS administered intraperitoneally directly after surgery. Mice were put back in cages in pairs of 2 and monitored closely. After 48 h, retro-orbital blood collection was performed, and mice were sacrificed by cervical dislocation. The right kidney was put in 4% formaldehyde for 24 h and then transferred to 70% ethanol for storage. Correspondingly, the left kidney was snap-frozen.

### Murine cisplatin-induced acute kidney injury

Eight- to twelve-week-old female mice were co-house at 2–5 mice/ cage in IVCs in our facility under 12 h light circles. As reported earlier [73], females were rigorously matched for weight, age, and genetic background to receive single dose cisplatin (20 mg/kg body weight if not otherwise indicated) intraperitoneally in a volume of 400 µl. After injection, mice were put back in cages at 2–5 animals and monitored closely. After 48 h, retro-orbital blood collection was performed, and mice were sacrificed by cervical dislocation. The right kidney was put in 4% formaldehyde for 24 h and then transferred to 70% ethanol for storage. Correspondingly, the left kidney was snap-frozen. For survival analysis, mice were closely monitored for up to 7 days. If prespecified parameters of severe animal deterioration were met, mice were considered dead and were sacrificed according to German animal protection laws.

### Histology and evaluation of structural organ damage

For histology, the kidneys were dehydrated in a graded ethanol series and xylene and finally embedded in paraffin. Paraffin sections (3–5 μm) were stained with periodic acid-Schiff (PAS) reagent, according to standard routine protocol. Stained sections were analyzed using an Axio Imager microscope (Zeiss) at ×100, ×200, and ×400 magnification. Micrographs were digitalized using an AxioCam MRm Rev. 3 FireWire camera and AxioVision ver. 4.5 software (Zeiss). Organ damage was quantified by two experienced pathologists in a double-blind manner on a scale ranging from 0 (unaffected tissue) to 10 (severe organ damage).

For the scoring system, tissues were stained with PAS, and the degree of morphological involvement in renal failure was determined using light microscopy. The following parameters were chosen as indicative of morphological damage to the kidney after ischemia-reperfusion injury (IRI): brush border loss, tubule dilatation, tubule degeneration, tubule necrosis, and tubular cast formation. These parameters were evaluated on a scale of 0–10, which ranged from not present (0), mild (1–4), moderate (5 or 6), severe (7 or 8), to very severe (9 or 10). The following parameters were chosen as indicative of morphological damage to the kidney after cisplatin injection: brush border loss, red blood cell extravasation, tubule dilatation, tubule degeneration, tubule necrosis, and tubular cast formation. These parameters were evaluated on a scale of 0–4, which ranged from not present (0), mild (1), moderate (2), severe (3), to very severe (4). Each parameter was determined on at least five different animals per group.

### Immunohistochemistry (IHC)

Organs (mouse or human) were dissected and transferred into 4% (vol/vol) neutral buffered formaldehyde, fixed for 24 h, and subsequently transferred to 70% ethanol. For histology, the kidneys were dehydrated in a graded ethanol series and xylene and finally embedded in paraffin. The sections (1.5 μm) were then deparaffinized, rehydrated, and boiled in citrate buffer pH 6 for 5 min (DC module 110 °C, Biocare). After 30 min cooling in a water bath, the slides were washed and treated with 3% H_2_O_2_ followed by blocking in 1% BSA for 1 h. Sections were then incubated o/n with anti-GSDMD (Abcam, ab219800) antibody (1:500 in 1% BSA) or anti CD3 (Dianova, DIA-P3E) antibody (1:200 in 1% BSA without antigen retrieval). The primary antibody was detected using a secondary goat anti-rabbit antibody (Abcam, ab214880; 1 h at room temperature) and the DAB substrate kit (Abcam ab64238; 5 min). After counterstaining with hematoxylin (1 min), sections were analyzed using an Axio Imager microscope (Zeiss) and AxioVision version 4.5 software (Zeiss). For negative controls, the primary antibody was omitted and replaced with a blocking solution.

### Mouse model of calcium oxalate-induced acute kidney injury

Male 8-week-old *Gsdmd*-deficient mice and WT mice received an oxalate-rich diet for 20 days. Oxalate-rich diet was prepared by adding 50 μmol/g sodium oxalate to a calcium-free standard diet (Ssniff, Soest, Germany). Mice were sacrificed by cervical dislocation on day 20 after initiation of the CaOx-enriched diet as described [74]. Plasma samples were collected, and GFR was measured at baseline and before sacrifice. Kidneys were harvested after sacrifice and kept in RNA later solution at −20 °C.

GFR measurements were performed in conscious mice on days 0 and 20 as described [75]. Therefore, mice were anesthetized with isoflurane to mount a miniaturized imager device built from two light-emitting diodes, a photodiode and a buttery (MediBeacon, Mannheim, Germany), onto the shaved neck of the animals. The background signal of the skin was recorded for 5 min. Subsequently, mice received an intravenous injection of 150 mg/kg FITC-sinistrin (MediBeacon, Mannheim, Germany). During the entire process, mice were conscious and kept in a single cage, and the signal was recorded for 90 min. Data were analyzed using MPD Lab software (MediBeacon, Mannheim, Germany). The GFR [μl/min] was calculated from the decrease of fluorescence intensity over time using a two-compartment model, the animals' body weight, and an empirical conversion factor [76].

### Real-time PCR

To assess mRNA expression levels, total RNA was isolated from kidneys using an RNA extraction kit (Invitrogen, USA) following the manufacturer’s instructions. RNA quality was assessed using agarose gels before transcription into cDNA using reverse transcriptase (Superscript II; Invitrogen, Carlsbad, CA, USA). Real-time quantitative PCR was performed using SYBRGreen PCR master mix, analyzed with Light Cycler 480 (Roche, Mannheim, Germany). Gene expression values were normalized using 18 s rRNA as a housekeeping gene. The primers used for amplification were purchased from Metabion (Martinsried, Germany) and are listed below.

**Table:** 

Target	Primer sequences
18 s	Forward: 5’-GCAATTATTCCCCATGAA-3’
	Reverse: 5’-AGGGCCTCACTAAACCAT-3’
NGAL	Forward: 5’-ATGTCACCTCCATCCTGG-3’
	Reverse: 5’-GCCACTTGCACATTGTAG-3’

### Isolation of fresh murine tubules

Primary murine renal tubules were isolated following a modified previously published protocol [38]. Briefly, murine kidneys were removed, washed with PBS, decapsulated, and sliced in four to five slices. Kidney slices of each kidney were transferred in 2 ml tubes containing 2 mg/ml collagenase type II in incubation solution (48 μg/ml trypsin inhibitor, 25 μg/ml DNAse I, 140 mM NaCl, 0.4 mM KH_2_PO_4_, 1.6 mM K_2_HPO_4_ × 3 H_2_O, 1 mM MgSO_4_ × 7 H_2_O, 10 mM CH_3_COONa × 3 H_2_O, 1 mM a-ketoglutarate and 1.3 mM Ca-gluconate) and digested for 5 min at 37 °C, 850 rpm. In order to minimize the presence of damaged tubules, the first resulting supernatant was discarded. Afterward, 1 ml of 2 mg/ml collagenase type II in incubation solution was added to the kidney slices and digested for 5 min at 37 °C, 850 rpm. The supernatant was collected and transferred in a 2 ml tube containing 1 ml ice-cold sorting solution (0.5 mg/ml bovine albumin in incubation solution). Tubes were left on ice for the tubules to precipitate. The supernatant was removed, and the tubules were washed twice with an ice-cold incubation solution. After precipitation of the tubules, the supernatant was removed, and an ice-cold sorting solution was added (volume was adjusted depending on the number of samples needed for the experiment). Tubules were distributed in a 24-well plate containing Dulbecco’s Modified Eagle Medium F-12 Nutrient Mixture without glycine and phenol red (DMEM/F-12, Thermo Fischer), supplemented with 0.01 mg/ml recombinant human insulin, 5.5 μg/ml human transferrin, 0.005 μg/ml sodium selenite (ITS without linoleic acid, Sigma–Aldrich), 50 nM hydrocortisone, 100 U/ml penicillin, and 100 μg/ml streptomycin (Pen/Strep, Thermo Fisher).

### Assessment of renal tubular cell death

LDH release of freshly isolated kidney tubules was measured according to manufacturers’ instructions at indicated time points. Briefly, an aliquot of the supernatant was taken, and Lysis Solution was added for 45 min to induce maximal LDH release before another aliquot of the supernatant was taken. Next, the supernatants were incubated with CytoTox 96® Reagent for 15 min protected from light at room temperature before adding Stop Solution. Absorbance was measured at 490 nm.

All performed experiments concerning isolated murine renal tubules contain a negative control to assess LDH release at 0 h of incubation as quality control. Experiments were considered valid when the negative controls had less than 10% LDH release. Isolated murine renal tubules were placed in 24-well plates containing the respective agents diluted in Dulbecco’s Modified Eagle Medium F-12 Nutrient Mixture without glycine and phenol red (DMEM/F-12, custom made by Cell Culture Technologies LLC, Switzerland), supplemented with 0.01 mg/ml recombinant human insulin, 5.5 μg/ml human transferrin, 0.005 μg/ml sodium selenite (ITS without linoleic acid, Sigma–Aldrich), 50 nM hydrocortisone, 100 U/ml penicillin, and 100 μg/ml streptomycin (Pen/Strep, Thermo Fisher). After indicated time points, the medium of each well was collected, and tubules were prepared for LDH release assay. Images of the treated murine renal tubules were obtained using a ×20/0.30 PH1 objective on a Leica DMi1 microscope.

### Statistics

Statistical analyses were performed using Prism 8 (GraphPad software, San Diego, CA, USA). To test the null hypothesis of no difference between groups in the survival experiments, we plotted the animals in a Kaplan–Meier curve and used the log-rank test for statistics. In all other experiments, two-tailed parametric *t*-test was used for normally distributed datasets, and one-way ANOVA followed by post hoc Tukey’s multiple comparisons test was used for analyzing data to compare multiple groups. Data were considered significant when *p ≤ 0.05, **p ≤ 0.01, or ***p ≤ 0.001. If not otherwise indicated, bar graphs represent the mean plus standard deviation.

### Reporting summary

Further information on research design is available in the Nature Research Reporting Summary linked to this article.

## Supplementary information

Supplementary figure descriptions

Fig.S1: Gasdermin D deposition following bilateral renal IRI.

Fig.S2: Investigation of renal tissue and function of Gsdmdko, Gsdmeko, and Gsdmd/Gsdmedko mice.

Fig. S3: Investigation of Gsdmd-deficient mice in a model of severe IRI

Fig. S4: Infiltration of CD3-positive cells is unchanged in Gsdmdko mice.

Fig.S5: Investigation of renal tissue and function of untreated Mlkl/Gsdmddko mice.

Fig. S6: The hypersensivity to cisplatin-induced AKI of Gsdmd-deficient, but not of Gsdme-deficient mice, depends on TNFα.

Fig. S7: Gsdmd-deficient mice are hypersensitive to calcium oxalate-induced AKI.

Reporting Summary

## Data Availability

The authors confirm that all data required to evaluate the conclusions of this study are presented in the paper and/or the Supplementary Materials. Other data are available from the corresponding author upon reasonable request.
