# Supplementary material for: Gasdermin D-deficient mice are hypersensitive to acute kidney injury
Source: Cell Death Dis. 2022 Sep 15;13(9):792. doi: 10.1038/s41419-022-05230-9 (PMC9478139; doi:10.1038/s41419-022-05230-9)
Supplement: Supplementary file 1 — Supplementary figure descriptions [file 41419_2022_5230_MOESM1_ESM.docx]

**Supplementary Information**

**Gasdermin D-deficient mice are hypersensitive to acute kidney injury**

Short title: The role of GSDMD in AKI

Wulf Tonnus^1,2^; Francesca Maremonti^1,2^; Alexia Belavgeni^1,2^; Markus Latk^1,2^; Yoshihiro Kusunoki^3^, Anne Brucker^1,2^, Anne von Mässenhausen^1,2^; Claudia Meyer^1,2^; Sophie Locke^1,2^; Florian Gembardt^1^, Kristina Beer^1,2^; Paul Hoppenz^1,2^, Jan U. Becker^4^; Christian Hugo^1^; Hans-Joachim Anders^3^ Stefan R. Bornstein^1, 5-8^; Feng Shao^9^ and Andreas Linkermann^1,2†^

^1^Department of Internal Medicine 3, University Hospital Carl Gustav Carus at the

Technische Universität Dresden, Dresden, Germany

^2^Biotechnology Center, Technische Universität Dresden, Dresden, Germany

^3^ Renal Division, Department of Medicine IV, University Hospital of the Ludwig Maximilian University, Munich, Germany

^4^Institute of Pathology, University Hospital of Cologne, Cologne, Germany.

^5^Diabetes and Nutritional Sciences, King's College London, London, UK.

^6^Center for Regenerative Therapies, Technische Universität Dresden, Dresden, Germany.

^7^Paul Langerhans Institute Dresden of Helmholtz Centre Munich at University Clinic Carl Gustav Carus of TU Dresden Faculty of Medicine, Dresden, Germany.

^8^Lee Kong Chian School of Medicine, Nanyang Technological University, Singapore, Singapore.

^9^National Institute of Biological Sciences (NIBS), Beijing, China.

**Fig.S1: Gasdermin D deposition following bilateral renal IRI.** High resolution micrographs presented in Fig. 1A, murine kidney from wild type and *Gsdmd*-ko following bilateral IRI, stained by immunohistochemistry for GSDMD.

**Fig.S2: Investigation of renal tissue and function of *Gsdmd^ko^*, *Gsdme^ko^*, and *Gsdmd*/*Gsdme^dko^* mice.** (**A**) Kidneys of 12-week-old male wildtype, *Gsdmd^ko^*, *Gsdme^ko^*, and *Gsdmd*/*Gsdme^dko^* mice were evaluated by PAS staining with no histological signs of tubular damage or any detectable phenotype. (**B-C**) Functionally, serum concentrations of creatinine and urea were indistinguishable from wild type littermates. (n = 3 mice/group) (ANOVA analysis, not significant for all groups tested)

**Fig. S3: Investigation of *Gsdmd*-deficient mice in a model of severe IRI.** 8 – 12-week-old male wildtype, *Gsdmd^ko^* mice underwent severe IRI and were sacrificed after 24 or 48 hours of reperfusion, respectively. (**A**) Histological evaluation revealed increased tubular damage in all groups investigated, quantified by tubular damage scores (**B**). Serum creatinine (**C**) and serum urea (**D**) concentrations upon sacrifice 24 or 48 hours after the onset of reperfusion are shown. Note the statistically insignificant comparison between the groups of wild type littermates and *Gsdmd^ko^* mice. (n = 3-9 mice/group, as indicated) (student´s t test for n.s. = not significant)

**Fig. S4: Infiltration of CD3-positive cells is unchanged in *Gsdmd^ko^* mice.** 8 – 12-week-old male wildtype and GSDMD^ko^ mice underwent moderate IRI and were sacrificed after 24 or 48 hours of reperfusion, respectively. Immunohistochemistry of CD3-positive cells (**A**) and quantification (**B**) revealed no statistically significant difference in all time points investigated. (n = 4-6 mice/group) (student´s t test for n.s. = not significant).

**Fig.S5: Investigation of renal tissue and function of untreated *Mlkl/Gsdmd^dko^* mice.** (**A**) Kidneys of 12-week-old male wildtype, *Gsdmd^dko^* and *Mlkl/Gsdmd^dko^* mice were evaluated by PAS staining with no histological signs of tubular damage or any detectable phenotype. (**B-C**) Functionally, serum concentrations of creatinine and urea were indistinguishable from wild type littermates. (n = 3-5 mice/group) (ANOVA not significant for all samples tested here)

**Fig. S6: The hypersensivity to cisplatin-induced AKI of *Gsdmd*-deficient, but not of *Gsdme*-deficient mice, depends on TNFα.** Female 8 – 12-week-old mice were treated with 20 mg/kg body weight of cisplatin i.p. (**A**) Survival curves of *Gsdme^ko^* mice are not different compared to wildtypes demonstrating the specificity of the GSDMD effect. (**B**) Kaplan-Meier-survival plot of indicated groups of mice following a single injection of 15 mg/kg BW cisplatin. Whereas TNFα-interference with etanercept prolonged survival in wild type mice, it did not in *Gsdmd^ko^* (n = 7-15 mice/group) (log-rank test with * p < 0.05).

**Fig. S7: *Gsdmd*-deficient mice are hypersensitive to calcium oxalate-induced AKI.** Male 8 – 12-week-old mice were fed upon calcium oxalate enriched diet as explained in the methods section. (**A**) Assessment of the glomerular filtration rate (GFR) in wild type and GSDMD-deficient mice following CaOx-enriched diet for 20 days (d20). (**B-C**) Serum concentrations of creatinine and urea (blood urea nitrogen, BUN) at 20d of CaOx-enriched diet. Note the non-significant trend to higher levels of the functional kidney markers. (**D**) mRNA expression of the tubular damage marker NGAL at 20d of CaOx-enriched diet. (n = 4-6 mice/group) (student t-test with * p < 0.05).
