# Supplementary figures and images for: Gasdermin D-deficient mice are hypersensitive to acute kidney injury
Source: Cell Death Dis. 2022 Sep 15;13(9):792. doi: 10.1038/s41419-022-05230-9 (PMC9478139; doi:10.1038/s41419-022-05230-9)

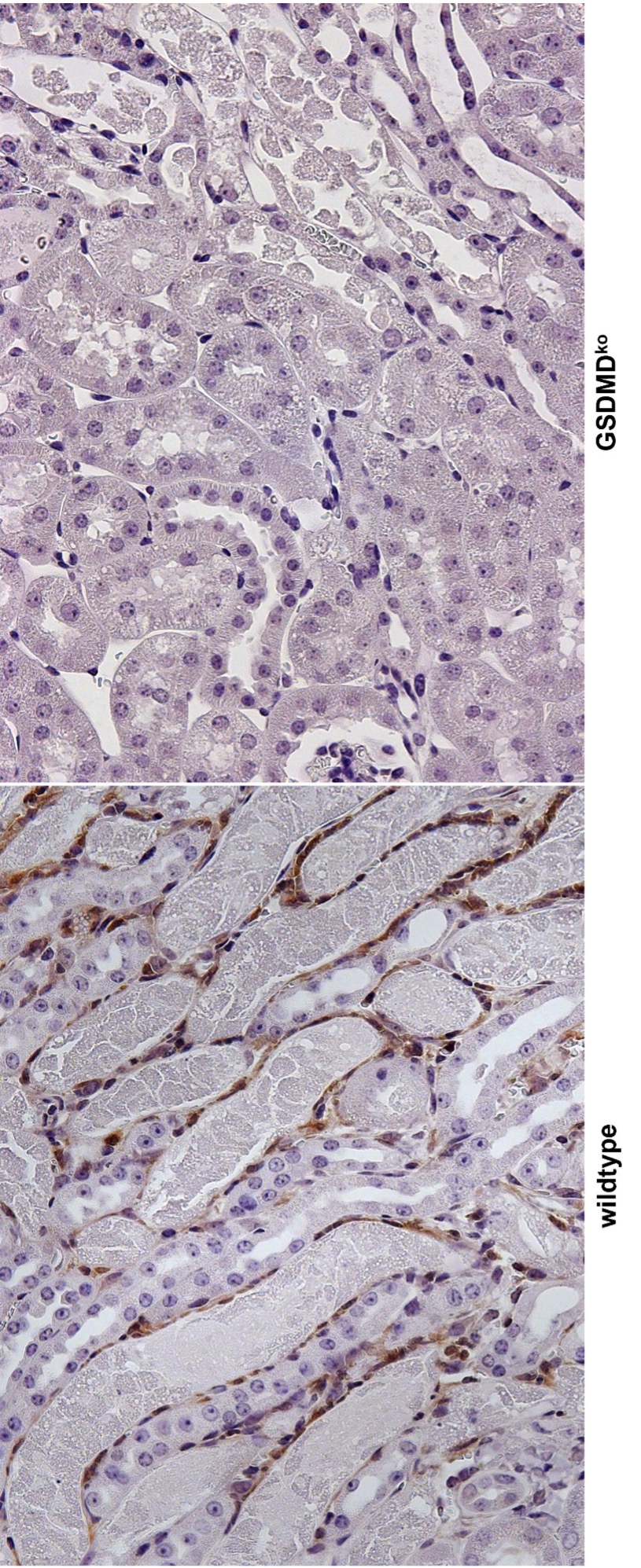

Supplement: Supplementary file 2 — Fig.S1: Gasdermin D deposition following bilateral renal IRI. [file 41419_2022_5230_MOESM2_ESM.jpg]

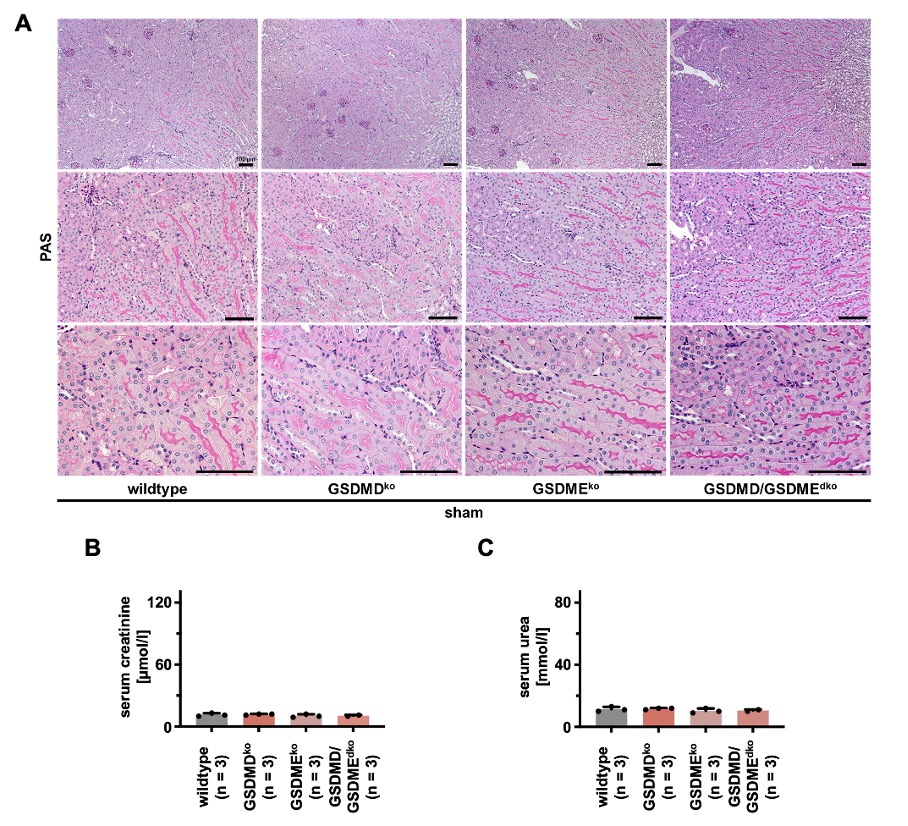

Supplement: Supplementary file 3 — Fig.S2: Investigation of renal tissue and function of Gsdmdko, Gsdmeko, and Gsdmd/Gsdmedko mice. [file 41419_2022_5230_MOESM3_ESM.jpg]

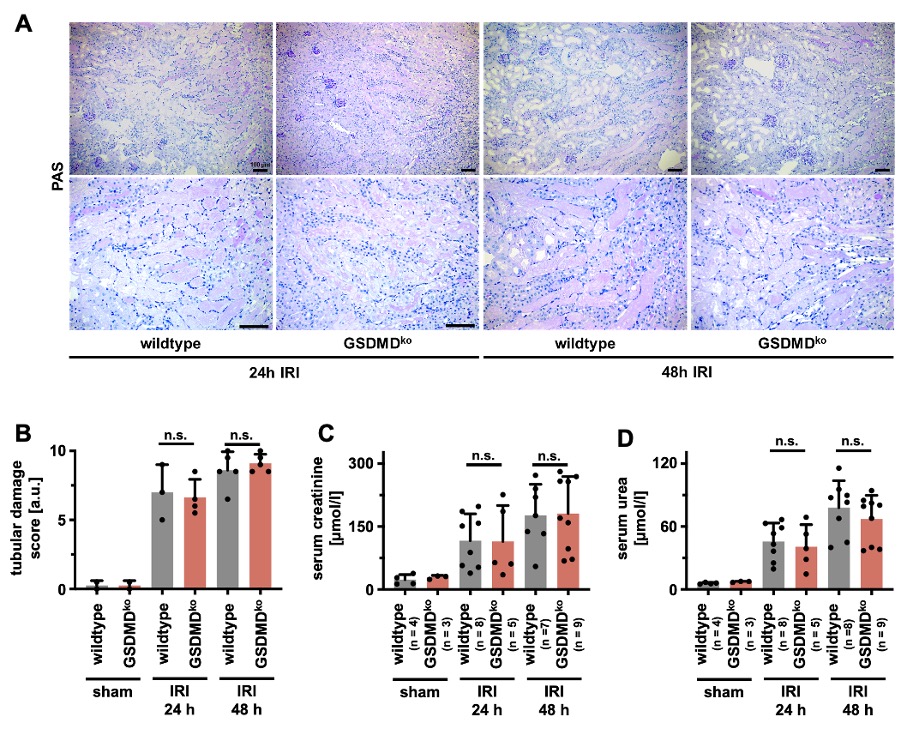

Supplement: Supplementary file 4 — Fig. S3: Investigation of Gsdmd-deficient mice in a model of severe IRI [file 41419_2022_5230_MOESM4_ESM.jpg]

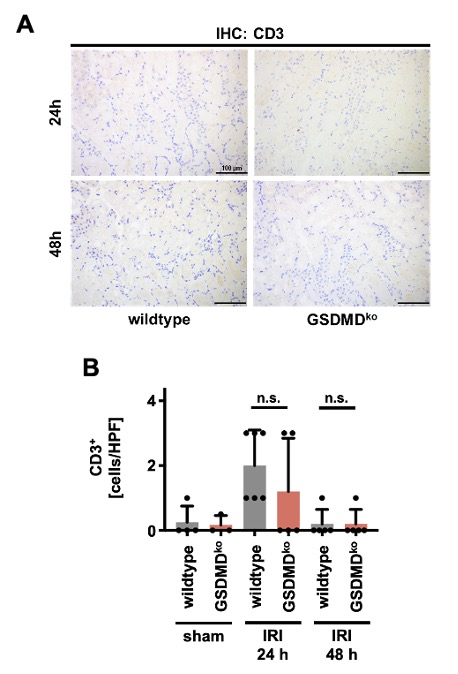

Supplement: Supplementary file 5 — Fig. S4: Infiltration of CD3-positive cells is unchanged in Gsdmdko mice. [file 41419_2022_5230_MOESM5_ESM.jpg]

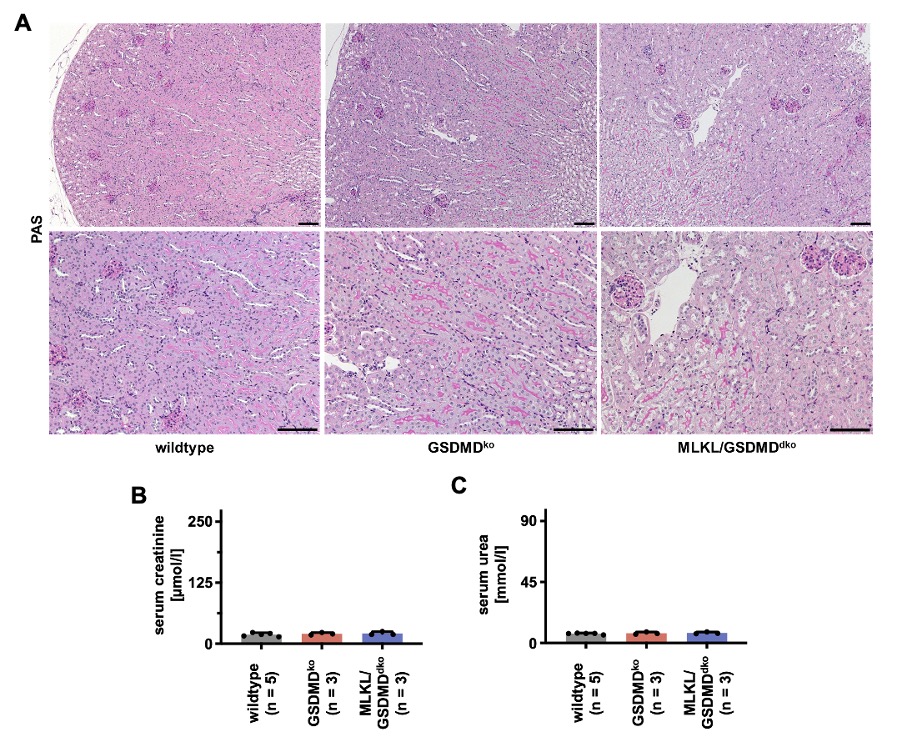

Supplement: Supplementary file 6 — Fig.S5: Investigation of renal tissue and function of untreated Mlkl/Gsdmddko mice. [file 41419_2022_5230_MOESM6_ESM.jpg]

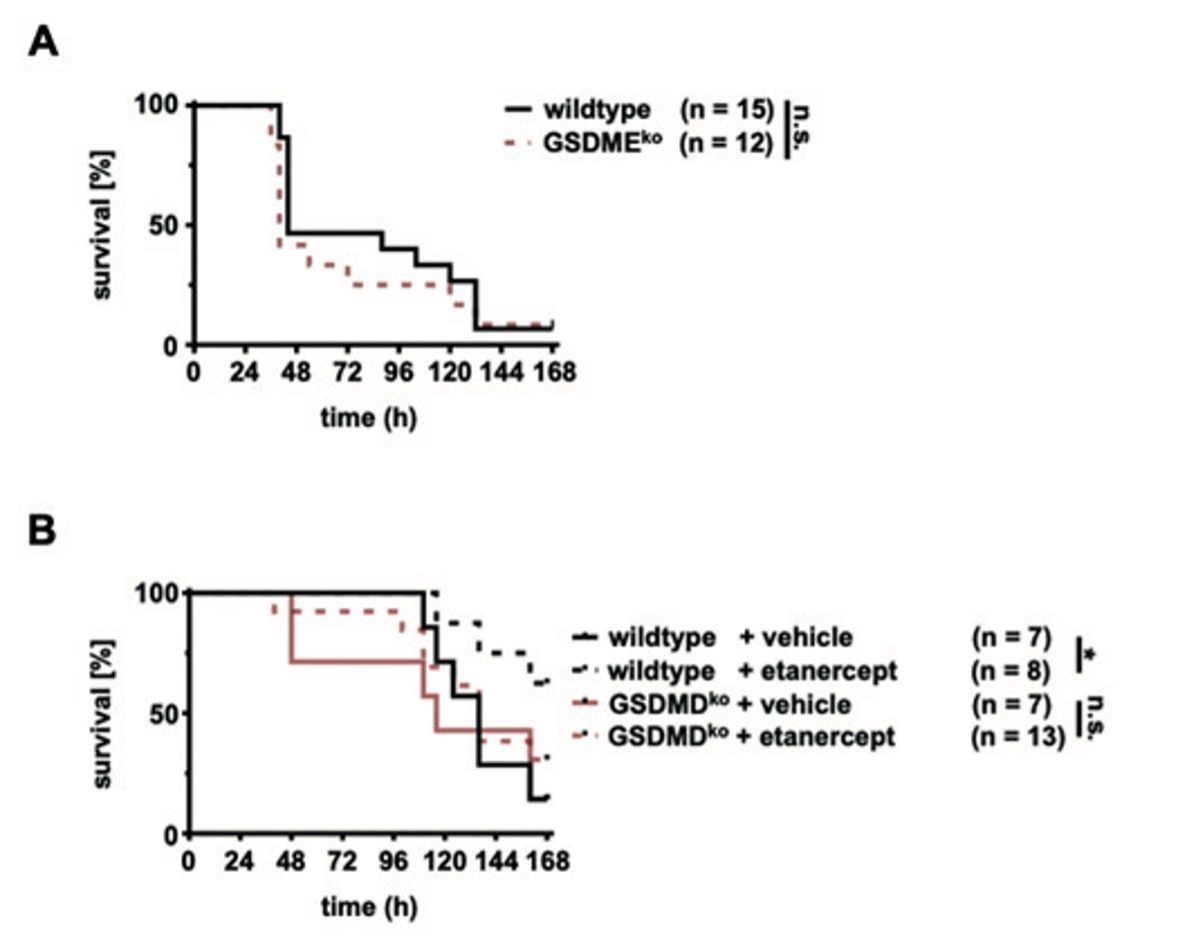

Supplement: Supplementary file 7 — Fig. S6: The hypersensivity to cisplatin-induced AKI of Gsdmd-deficient, but not of Gsdme-deficient mice, depends on TNFα. [file 41419_2022_5230_MOESM7_ESM.jpg]

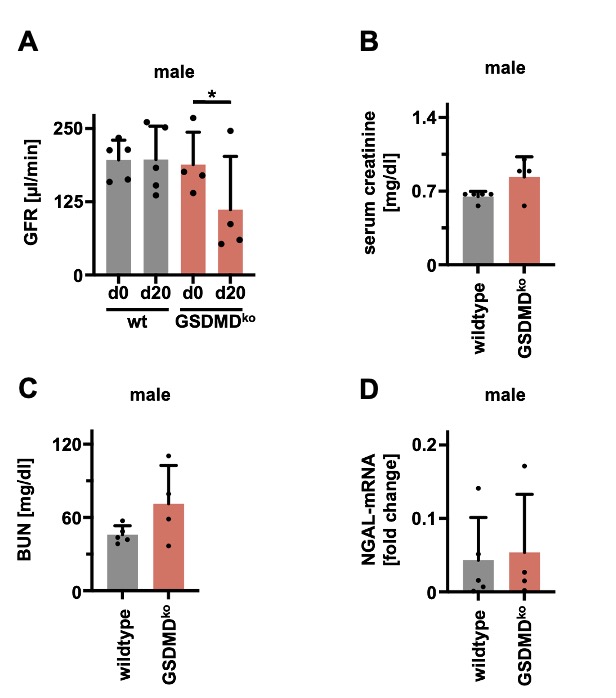

Supplement: Supplementary file 8 — Fig. S7: Gsdmd-deficient mice are hypersensitive to calcium oxalate-induced AKI. [file 41419_2022_5230_MOESM8_ESM.jpg]
